# Concise Review: Hematopoietic Stem Cell Origins: Lessons from Embryogenesis for Improving Regenerative Medicine

**DOI:** 10.5966/sctm.2016-0110

**Published:** 2016-08-02

**Authors:** Adriana De La Garza, Arpan Sinha, Teresa V. Bowman

**Affiliations:** ^1^ Department of Developmental and Molecular Biology, Albert Einstein College of Medicine, Bronx, New York, USA; ^2^ Gottesman Institute for Stem Cell Biology and Regenerative Medicine, Albert Einstein College of Medicine, Bronx, New York, USA; ^3^ Division of Pediatric Hematology/Oncology, Children's Hospital at Montefiore, Bronx, New York, USA; ^4^ Department of Medicine (Oncology), Albert Einstein College of Medicine, Bronx, New York, USA

**Keywords:** Hematopoietic stem cells, Developmental biology, Bone marrow transplant, Embryo

## Abstract

Hematopoietic stem cells (HSCs) have extensive regenerative capacity to replace all blood cell types, an ability that is harnessed in the clinic for bone marrow transplantation. Finding appropriate donors remains a major limitation to more extensive usage of HSC‐based therapies. Derivation of patient‐specific HSCs from pluripotent stem cells offers great promise to remedy this problem if scientists could crack the code on how to make robust, transplantable HSCs in a dish. Studies delving into the native origins of HSC production during embryonic development should supply the necessary playbook. This review presents recent discoveries from animal models, with a focus on zebrafish, and discusses the implications of these new advances in the context of prior knowledge. The focus is on the latest research exploring the role of epigenetic regulation, signaling pathways, and niche components needed for proper HSC formation. These studies provide new directions that should be explored for de novo generation and expansion of HSCs for regenerative therapies. Stem Cells Translational Medicine *2017;6:60–67*

**Significance Statement**

Hematopoietic stem cells (HSCs) have extensive regenerative capacity to replace all blood cell types, an ability that is harnessed in the clinic for bone marrow transplantation. Finding appropriate donors remains a major limitation to more extensive usage of HSC‐based therapies. Derivation of patient‐specific HSCs from pluripotent stem cells offers great promise to remedy this problem if scientists could crack the code on how to make HSCs in a dish. This review presents data from studies delving into the native origins of HSC production during embryonic development that provide new directions that should be explored for de novo generation and expansion of HSCs for regenerative therapies.

## Introduction

Hematopoiesis, the process of blood production, is maintained throughout the lifetime of an organism by long‐lived hematopoietic stem cells (HSCs) that are capable of self‐renewing and generating all types of mature blood cells needed to carry oxygen, fight infection, and prevent bleeding. Clinically, the success of bone marrow and cord blood transplantation therapies is because of the ability of HSCs in those tissues to repopulate the blood system of the new host. Limitations in the number of suitable donors for HSC‐based therapies greatly hamper broader usage of transplantation. Generation of patient‐specific HSCs from pluripotent stem cells holds great promise to remedy this problem if researchers could faithfully derive fully functional, transplantable HSCs in a dish. Although HSCs are present in adults, they are only generated de novo during embryonic development. Although our understanding of the origins of bona fide HSCs is still quite primitive, great strides have been made over the past several decades using animal models to uncover many novel and surprising steps in HSC ontogeny.

**Figure 1:**
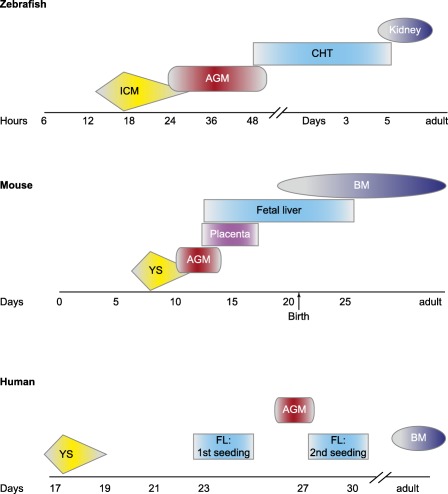
Comparison of timing of hematopoietic development across vertebrates. Time lines showing when and where primitive and definitive hematopoietic induction occurs in zebrafish (top), mice (middle), and humans (bottom). Abbreviations: AGM, aorta‐gonad‐mesonephros; BM, bone marrow; CHT, caudal hematopoietic tissue; FL, fetal liver; ICM, intermediate cell mass; YS, yolk sac.

Embryonic hematopoiesis occurs in sequential waves, which can be divided into primitive and definitive 1. Primitive waves form erythroid and myeloid cells to help the developing embryo through its first growth steps. The definitive waves generate erythromyeloid progenitors and long‐term HSCs 2 3 4. Generation of definitive HSCs occurs in a region termed the aorta‐gonad‐mesonephros (AGM). Within this region, HSCs specifically arise from specialized hemogenic endothelium found in the ventral wall of the dorsal aorta (DA) in a process termed the endothelial‐to‐hematopoietic transition that is conserved across vertebrates, including zebrafish (*Danio rerio*), mice, and humans (Fig. 1) 3, 5 6 7. After their specification, HSCs travel to an intermediate niche (fetal liver/placenta in mammals and caudal hematopoietic tissue [CHT] in zebrafish), where they proliferate and expand, and then ultimately colonize their final home, where they will remain throughout adulthood (bone marrow in mammals and kidney marrow in zebrafish) 1, 8 9 10. At each stage, the niche sends environmental signals that regulate HSC behaviors, including proliferation and differentiation 1.

Hematopoiesis is highly conserved among vertebrates, and most of our knowledge comes from animal models. Françoise Dieterlen‐Lièvre was among the first scientists to demonstrate the intraembryonic origins of definitive hematopoiesis within the DA through studies using chick‐quail chimeras 11. Later work confirmed that mice, *Xenopus*, and zebrafish form HSCs in a highly similar fashion 3, 12, 13.

Zebrafish provide an excellent model for the study of hematopoiesis because of its numerous advantages. External embryonic development allows for easy access to early embryonic stages, and embryonic transparency permits easy visualization. This latter characteristic allowed scientists to directly visualize in vivo HSC emergence in real time, revealing without a doubt the endothelial origins of HSCs 3, 6. In addition, rapid embryonic development, numerous genetic manipulation tools, and fluorescent transgenic animals make zebrafish highly amenable for exploring the ontogeny of HSCs. Recent studies in zebrafish have yielded novel findings in HSC specification, including contributions from the Notch signaling pathway, inflammatory signaling, and the HSC niche. This review summarizes these new discoveries and how they contribute to our knowledge of definitive hematopoiesis.

## Insights Into the Early Stages of HSC Specification

**Figure 2:**
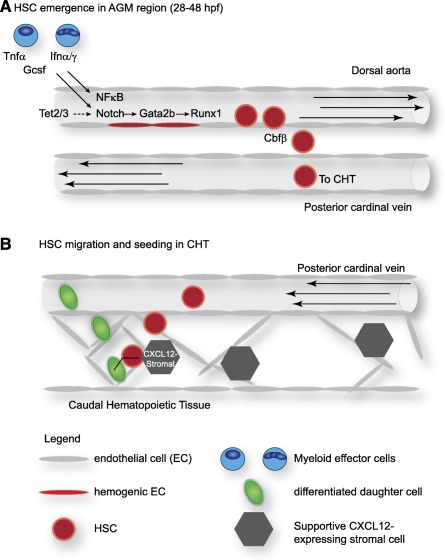
Diverse signals regulate embryonic HSC behaviors in zebrafish. **(A):** Inflammatory signals from myeloid effector cells promote HSC emergence through activation of Notch and NFκB signaling. Tet2/3 also regulate Notch signaling, leading to *gata2b* and *runx1* expression in hemogenic endothelial cells. Cbfβ is subsequently required to promote the extravasation of emerging HSC out of the dorsal aorta. **(B):** Nascent HSCs seeding the CHT induce endothelial remodeling to form a “microniche” comprising an HSC surrounded by endothelial cells adjacent to a CXCL12‐expressing supportive stromal cell. The orientation of the division plane of the HSC is dictated by the position of the stromal cell. An open arrow on the HSC (red) and daughter cell (green) shows the angle of the division plane. Arrows within the vessels show the direction of blood flow. Abbreviations: AGM, aorta‐gonad‐mesonephros; CHT, caudal hematopoietic tissue; EC, endothelial cell; hpf, hours postfertilization; HSC, hematopoietic stem cell; Ifnα/γ, interferon α/γ; Tnfα, tumor necrosis factor α.

The earliest HSC precursors arise from the posterior lateral plate mesoderm (PLM) that migrates to the medial region of the embryo and gives rise to a vascular cord that will become the dorsal aorta 14 15 16. Once the DA is formed and specified, HSCs emerge from specialized hemogenic endothelial cells that reside within its ventral floor. Once these cells take on a hematogenic fate, they bud out of the aorta, enter circulation, and seed the next developing niche (Fig. 2) 3, 6, 8. HSC specification and emergence require integration of intrinsic factors with input from different signaling pathways that interact to guide tissue development from the earliest mesoderm precursors until the final mature HSCs. Some of the principal signaling pathways that regulate HSC emergence identified from experiments in animal models are the vascular endothelial growth factor (VEGF) pathway, which stimulates endothelial cell differentiation and migration; Sonic hedgehog (SHH) and bone morphogenetic protein (BMP) signaling, which regulate arterial wall polarization necessary for HSC specification; and the Notch pathway 17 18 19. The Notch pathway is involved in many defining steps throughout HSC formation and specification that will be discussed in greater detail later in this review 19.

Transcriptional master regulators that coordinate with and sometimes instruct the epigenetic landscape are essential to define cellular fate. Key transcription factors for HSCs are Gata2, Scl, Runx1, Lmo2, and C‐myb 20. Despite our knowledge of these factors, their precise role in each step of HSC formation is still open to debate.

GATA2 has been well studied in hematopoiesis and is known to act downstream of Notch signaling during HSC specification 21. The importance of GATA2 in hematopoiesis was first demonstrated in mice, where it was shown that null embryos died at approximately embryonic day 10.5 with severe primitive and definitive hematopoietic defects 22. Moreover, studies of endothelial‐ and hematopoietic‐specific mouse knockouts of *Gata2* demonstrated a requirement for GATA2 both in the endothelial‐to‐hematopoietic transition and in HSC maintenance 23. Because GATA2 has a function in vasculature, it could act in a cell‐autonomous and/or a non‐cell‐autonomous fashion to regulate the endothelial‐to‐hematopoietic transition. Unlike mammals, zebrafish have two *gata2* genes, *gata2a* and *gata2b*. During evolution, the various functions of Gata2 became divided between these paralogs: Gata2a plays a role in vasculature formation, whereas Gata2b is required for HSC formation 24. This separation of function in Gata2 paralogs allows more refined dissection of the genetic control of Gata2 during vessel formation and HSC specification. In a recent study examining zebrafish *gata2b* function, Butko et al. found that hemogenic induction can be detected earlier during embryonic development than previously appreciated 24. *gata2b* expression starts in the PLM around the midline at 18 hours postfertilization (hpf) (14–19 somites) before the formation of the vascular cord, is later detected in the ventral wall of the DA at 25 hpf, and persists in hematopoietic cells in the CHT at 72 hpf. This novel finding opens the door to studying the earliest steps of hemogenic endothelium before DA formation.

Runx1 is another transcription factor that is indispensable for HSC formation, acting downstream of Notch signaling under the control of *gata2b* 19, 25. Runx1 interacts with the protein core binding factor subunit β (Cbfβ), and it is thought that they function together during HSC formation because knockout mice for both proteins show similar hematopoietic defects 26 27 28. New data from Bresciani et al. challenge the assumption that Runx1 and Cbfβ coordinate during HSC emergence 29. Their study in *cbfb*‐null mutant zebrafish showed that, like *runx1*, *cbfb* expression responds to Notch1 signaling. However, their work demonstrates that Cbfβ has a separate role from its partner Runx1 during HSC development. Similar to *runx1* mutants, the ultimate outcome in *cbfb* mutants is the lack of definitive hematopoiesis, but the stage of development where the defect occurs in the two mutants is distinct. Zebrafish *runx1* mutants fail to induce hematopoietic gene expression at early stages of HSC formation, and therefore HSCs fail to specify. Loss of *cbfb* does not affect initial HSC formation, but, rather, impairs their ability to detach from the DA and enter circulation (Fig. 2A). Further pharmacological studies inhibiting Runx1‐Cbfβ interactions confirmed that the role of both proteins during HSC development could be uncoupled. This study implied that both Runx1 and Cbfβ are needed at different times during HSC development: Runx1 acts during specification, and Cbfβ acts afterward at the time of HSC extravasation from the DA. The door remains open regarding alternative transcription factor partners for Runx1 and Cbfβ during HSC ontogeny.

Epigenetic factors add an additional layer of complexity to gene expression and cell state control. One epigenetic process that is critical for HSCs is DNA methylation. The Tet family of methylcytosine dioxygenases, comprising Tet1, Tet2, and Tet3, convert 5‐methylcytosine (5‐mC) (typically a mark of repressed gene expression) to 5‐hydroxymethylcytosine (5‐hmC), ultimately leading to DNA demethylation and changes in gene expression. Proper regulation of the Tet family proteins is required for normal adult hematopoiesis. Mutations in *TET1* and *TET2* are prevalent in leukemias and myeloid malignancies 30, 31. In addition, loss of either TET1 or 2 in mice leads to clonal hematopoiesis, a precursor to leukemia 32 33 34. Despite this knowledge, the role of Tet factors in embryonic hematopoiesis was mostly unknown. Li et al. recently determined that Tet2 and Tet3 are redundantly required during HSC emergence 35. Zebrafish embryos mutant for both *tet2* and *tet3*, but not either single mutant, showed diminished production of HSCs. The *tet2/3* double mutants have diminished *gata2b* expression, and restoring *gata2b* RNA to embryos rescued HSC formation (Fig. 2A). Further analysis revealed that Notch signaling was decreased specifically in the ventral wall of the DA, suggesting that DNA methylation has a particularly important role in Notch pathway regulation during the endothelial‐to‐hematopoietic transition. This study showed that the Tet proteins are important for both embryonic and adult hematopoiesis, expanding our current understanding of developmental epigenetic regulation. It is also interesting to note that Tet redundancy allows individual mutants to develop normally, which could be interpreted as genetic protection of the important process of HSC formation.

## Muscling Up HSCs

**Figure 3:**
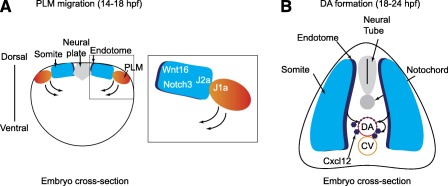
Somitic signals regulate hematopoietic stem cell (HSC) formation in zebrafish. **(A):** Cross‐sectional view of a zebrafish embryo at 14 hpf. The posterior lateral mesoderm migrates medially, sliding under the somites, where it receives Wnt16 and Notch3 signals that promote hematopoietic induction. The close proximity of PLM and somite cells is achieved by heterotypic interactions between Jam1a on PLM and Jam2a on somites, which helps to strengthen the Notch signal. **(B):** Cross‐sectional view of a zebrafish embryo at 24 hpf. Anterior portions of somites give rise to the endotome, which contributes cells to the dorsal aorta that promote HSC formation, in part by Cxcl12 production. Curved arrows denote the movement of cells. Abbreviations: CV, caudal vein; DA, dorsal aorta; hpf, hours postfertilization; J1a, Jam1a; J2a, Jam2a; PLM, posterior lateral mesoderm.

As mentioned before, hemogenic endothelium, nonhemogenic vasculature, and HSCs all originate from the PLM (also known as splachnic mesoderm) 14, 15. In zebrafish, it was thought that all cells of the aorta arose from this splachnic mesoderm, but new data challenged this dogma. Nguyen et al. recently showed that somite‐derived endothelial cells contribute to DA formation 36, similar to what happens in mouse and chick 37. These experiments revealed that the somitic contribution comes from the endotome, a specific central region within somites specified by the homeobox factor Meox1 (Fig. 3) 36. These cells are in fact key players in HSC induction: through chemokine signaling, endotome‐derived cells can induce HSC fate in their DA neighbors. Because somitic‐derived endothelial cells are also present in the mammalian DA, it will be interesting to see whether this mechanism is conserved in mammals.

Previous studies in zebrafish implicated a role for muscle‐derived signals in HSC formation. Clements et al. demonstrated that expression of the Notch ligands *deltaC* (*dlc*) and *deltaD* (*dld*) in somites is necessary for HSC specification 38. However, the details of the interactions between somites and precursor hemogenic cells were not known. Recent work from Kobayashi et al. established that HSC fate begins during PLM migration 39. They found that the migrating PLM and the somites they move across are each expressing partner junctional adhesion molecules: PLM cells express *jam1a*, whereas somite cells express *jam2a*. Jam1a and Jam2a interact, allowing both types of cells to have close physical contact with each other, which is required for proper intercellular Notch signal transduction (Fig. 3).

This novel involvement of muscle in HSC specification illustrates the dependence of HSC emergence on neighboring tissues. The works from Nguyen et al. 36 and Kobayashi et al. 39 show without a doubt that interactions between embryonic muscle and endothelium are more complex than previously thought and are indispensable for HSC formation. Furthermore, the Kobayashi et al. 39 findings are striking because they also demonstrate that hemogenic endothelium and HSC fate are acquired much earlier than previously thought, in agreement with the work by Butko et al. 24. It seems that the stage of PLM migration is of great importance for HSC specification, and more research focusing on this developmental window is greatly warranted.

## Notch at the Many Crossroads of HSC Birth

Notch signaling occurs between adjacent cells through interaction between ligands and receptors. In mammals, four receptors (Notch1–Notch4) and five canonical ligands (Jagged1, Jagged2, delta‐like 1 [Dll1], Dll3, and Dll4) have been identified. Upon ligand binding, the intracellular domain of the Notch receptor (NICD) is cleaved and translocates to the nucleus, where it acts as a transcription factor 40.

Signaling via the Notch pathway is critical for multiple steps of HSC formation, from induction of endothelial tissue to HSC emergence 19. It acts early during development promoting the determination and proliferation of endothelial precursors within the PLM, and later it directly regulates expression of genes acting more proximal to HSC formation, such as *gata2*, which in turn drives *runx1*, a necessary step for HSC emergence 19. In addition, somitically expressed ligands Dld and Dlc are required to induce hemogenic fate in endothelial cells 38, 39.

Of the four mammalian receptors, NOTCH1 was shown to be required cell‐autonomously for HSC specification, whereas NOTCH2 seems to be dispensable 41. However, the rest of the receptors were not thoroughly characterized in murine HSC development. Recently, Kim et al. analyzed the role of four Notch receptors (the *Notch1* paralogs *notch1a* and *notch1b*, *notch2*, and *notch3*) during HSC induction 42. In agreement with the murine studies, zebrafish Notch1a and Notch1b are required cell‐autonomously in the endothelium (approximately 20 hpf) for HSC formation. Zebrafish Notch2, like murine NOTCH2, is dispensable for HSC specification, suggesting that Notch receptor functions are conserved in vertebrates. A novel finding from this work is that Notch3 signaling is required for HSC specification, but in a non‐cell‐autonomous fashion. The *notch3* gene is expressed in PLM and somites early during development at 13 hpf and then becomes restricted to endothelium including the DA at 24 hpf. Surprisingly, it is somitic Notch3, and not endothelial Notch3, that is required for HSC formation at approximately 14 hpf (Fig. 3). It also appears that Notch3 is acting downstream of the previously known somitic Notch ligands Dlc and Dld. All together, this body of work has given us tremendous insight into the specific roles of different Notch receptors, showing that they perform nonredundant functions in muscle, endothelium, and HSCs during HSC formation, and that their roles are temporally and spatially regulated.

The complex role of Notch in HSC induction is highly conserved in mammals. Jang et al. demonstrated that overexpression of the Notch1 intracellular domain in murine embryonic stem cells resulted in increased hematopoietic differentiation during embryoid body differentiation 43. Microarray analysis of gene expression after induction of NICD overexpression allowed identification of several upregulated genes that could be involved in the specification of hemogenic endothelium, including the transcription factor *Foxc2*, previously known to be involved in arterial specification 44. Subsequent in vivo knockdown of the *Foxc2* paralogs *foxc1a* and *foxc1b* in zebrafish embryos showed a loss of HSCs that was not rescued by NICD overexpression, suggesting that Foxc2 acts downstream of Notch. *Foxc2*‐null mice also have defective HSC formation, suggesting that the requirement of FOXC2 for definitive hematopoiesis is conserved in vertebrates. The discoveries from this study offer vital pieces of information on vertebrate Notch signaling and are especially valuable for improving the current state of in vitro HSC generation for future clinical applications.

## Inflammatory Signaling

Studies in the last few years have shown that HSCs have a role in the early immune response to infection 45 46 47 48 49. HSCs proliferate in response to systemic infection to replace effector immune cells 50. This response is not simply secondary to diminished downstream immune cell numbers, but, rather, a direct response to systemically elevated cytokines signaling through cytokine receptors on HSCs 51. It was also found that HSCs could respond to bacterial and viral components directly through toll‐like receptors (TLRs) via the TLR‐nuclear factor κ‐light chain enhancer of activated B (TLR‐NFκB) axis 48, 49, 52.

More recently, similar cytokine signaling pathways have been found to play a critical role during embryonic HSC production independent of infection, a process termed sterile inflammation 53 54 55 56 57. The innate immune system is established early during primitive hematopoiesis with the formation of the myeloid effector cells neutrophils and macrophages. The molecular effectors produced by these cells are various cytokines, including tumor necrosis factors (TNFs) like TNF‐α and ‐β, interleukins (ILs) like IL‐6, and interferons (IFNs) like IFN‐α and ‐γ. Various studies in the last few years have explored a critical role for these proinflammatory cytokines in HSC development.

Through expression profiling studies, Li et al. showed that HSCs from the AGM regions of midgestation mouse embryos have a robust innate inflammatory gene signature 55. In particular, they observed a strong IFN signature, hinting that embryonic HSCs are responding to inflammatory cues. Combined studies in murine and zebrafish systems further demonstrated that IFN signaling is important for HSC development. Mouse or zebrafish embryos lacking IFN‐α or ‐γ signaling had significantly fewer AGM HSCs 55 (Fig. 2A). Sawamiphak et al. made a similar observation demonstrating that zebrafish lacking Ifn‐γ or its receptor Crfb17 act cell‐autonomously to positively regulate HSC development by modulating the endothelial‐to‐hematopoietic transition 56.

Other groups found supportive roles for other proinflammatory cytokines. TNF‐α signaling was shown to be important for the development of embryonic vasculature and HSCs 53, 58. By using zebrafish mutants and morpholino‐mediated knockdown, it was shown that depletion of *tnfa* and its receptor *tnfr2* resulted in reduced numbers of HSCs in the floor of the dorsal aorta, which was not due to a secondary effect of disrupted vascular development 53, 54. Combined knockdown of *tnfa* and *ifn‐γ* resulted in more severe reduction of *runx1*‐positive HSCs than loss of either cytokine individually, suggesting nonredundant and cooperative functions of multiple proinflammatory cytokines during HSC development 55.

Granulocyte‐colony‐stimulating factor (Gcsf) signaling to endothelial cells was first demonstrated during embryogenesis by Liongue et al. where they showed that Gcsf/Gcsfr signaling was required during the emergency granulopoietic response in embryos 59. Stachura et al. recently demonstrated a role for Gscf in HSC emergence and expansion in zebrafish embryonic development 57. This cytokine was also recently implicated to act in the inflammatory signaling pathway during HSC development 54. Tlr4bb and its downstream effectors Myd88 and NFκB act downstream of both Gcsf and Tnf‐α within hemogenic endothelial cells to promote HSC production 54 (Fig. 2A).

As previously discussed, Notch is a well‐established pathway controlling HSC formation 38, 39, 41 42 43, 60. In the above studies, Notch was found to be a common effector of proinflammatory signaling 53, 54, 56. Tnf‐α/Tnfr2 signaling activates Notch by inducing expression of the Notch ligand *jagged1a* (*jag1a*) in endothelial cells in the DA 53. Jag1a then activates the Notch1a receptor on the neighboring hemogenic endothelium resulting in activation of the NFκB transcription factor and subsequent promotion of the endothelial‐to‐hematopoietic transition 53. In contrast, Ifn‐γ acts downstream of Notch signaling through atypical activation of Stat3 in the DA 56.

Based on the above studies, it is clear that sterile inflammatory signaling has a fundamental role in HSC emergence. Interestingly, the cellular source of cytokine production important for HSC emergence appears to be the neutrophils and macrophages generated from the first wave of embryonic hematopoiesis, indicating a new level of dependency of definitive HSCs on primitive hematopoietic cells 53 54 55, 61. Work is still needed to resolve the precise molecular and cellular mechanisms used by each myeloid cell type and each cytokine signaling pathway during HSC emergence. Recapitulation of this complex in vivo milieu is likely necessary to improve production of HSCs in vitro.

## Defining the Developmental Niche

Ray Schofield was among the first to propose the concept of the niche as a place where HSCs reside 62. The niche that we now know is not only anatomic, but has a large functional dimension 63, 64. HSC niches are specialized microenvironments that maintain and regulate the HSCs, modulating their proliferation, differentiation, and self‐renewal. The interplay between HSCs and their niche is complex and dynamic, involving cell‐to‐cell interactions and secreted growth factors and chemokines, as well as physical parameters. During development, HSCs travel between niches to establish hematopoiesis (Fig. 1). After arising from hemogenic endothelium in the dorsal aorta, HSCs seed an intermediate hematopoietic niche—fetal liver in mammals and caudal hematopoietic tissue in zebrafish 65. HSCs expand rapidly in this intermediate niche and then move out and seed the adult marrow—found within bone in mammals and kidney in zebrafish. The adult niche in mice is well defined and comprises many different components, including mesenchymal stem cells, endothelial cells, osteoblasts, arterioles, and sympathetic nerves, as well as some hematopoietic elements, including megakaryocytes and macrophages 63. The secrets of developmental niches are largely elusive, but recent advances are beginning to shine some light on the problem.

To uncover the molecular underpinnings of various developmental niches, Charbord et al. recently completed a comprehensive transcriptome meta‐analysis of HSC‐supportive cells and defined the core molecular networks of the niche from the embryo to adults 66. The data support the view that niches have different characteristics emblematic of the needs of their tissue of origin. They showed that the bone marrow‐supportive cells exhibited a mesenchymal phenotype, AGM‐supportive cells expressed genes implicated in blood vessel formation, and fetal liver‐supportive cells expressed genes related to cell cycle regulation 66. These data highlight the plastic nature of niche cells and the influence of the environment on their function.

Because of the complexity of cell types that comprise the in vivo niche, cell culture systems have been invaluable to defining which cell types are capable of supporting HSC maintenance, expansion, or differentiation. Experiments using explant cultures of AGM regions helped to define the importance of BMP, VEGF, and SHH signaling from this development niche 37, 67, 68. The zebrafish is an established in vivo model, but new developments in zebrafish cell culture are now providing similar tools to mammalian systems that will permit the discovery of HSC niche components 69, 70. These discoveries can then be easily tested for functionality in vivo. Campbell et al. recently described the creation of a stromal line, zebrafish embryonic stromal trunk (ZEST) cells, which are derived from tissue surrounding the embryonic dorsal aorta 71. Similar to the findings from Charbord et al. 66 regarding mammalian AGM niche cells, ZEST cells have endothelial characteristics. Through the expression of various cytokines, ZEST cells can support both HSC proliferation and multilineage differentiation 71. This model will be a useful tool in studying the niche components important for HSC emergence and differentiation.

The next developmental niche in zebrafish is the caudal hematopoietic tissue—the functional equivalent to the mammalian fetal liver—where HSCs expand and differentiate. Recent work from Tamplin et al. explored the spatial relationship between HSCs, endothelial cells, and stromal cells within the CHT 72. They found that HSCs trigger a dynamic remodeling of endothelial cells during colonization of the CHT 72. When an HSC arrives in the perivascular niche of the CHT, a group of endothelial cells remodels to form a surrounding pocket (Fig. 2B). During cell division, the HSC is then anchored to a perivascular stromal cell, which dictates the orientation of the division plane. Interestingly, they observed similar cellular behaviors of HSCs and niche components in the murine fetal liver, indicating that this process is highly conserved. They also performed a chemical genetic screen in zebrafish to identify molecules that control the process of HSC engraftment into the CHT. They identified lycorine as a novel compound that promoted HSC lodgement during development and ultimately led to a sustained increase in the size of the stem cell pool into adulthood 72. Improving HSC lodgement after bone marrow transplantation with lycorine could represent a new therapeutic modality for improving engraftment and patient health after transplantation.

Together, these studies demonstrate the changing form and function of each developmental niche. Understanding how the AGM niche promotes HSC specification can lead to improvements in HSC production from pluripotent stem cells, while studies of how the CHT/fetal liver niche promotes HSC proliferation can aid in the expansion of HSCs in vitro to improve current bone marrow transplantation approaches. Extension of the above approaches to understand the development of the bone and kidney niches will provide insight into the origins of the ultimate HSC niche. Insights into how microenvironments adjust to the requirements of their HSC residents could reveal new therapies for treating diseases such as leukemia and improving outcomes in stem cell transplantation settings.

## Conclusion

Our understanding of the origins and regulation of definitive hematopoietic stem cells has come a long way since the groundbreaking work of Dieterlen‐Lièvre and others. In particular, recent advances have identified new cellular constituents that were heretofore unknown to constitute the developing microenvironment important for HSC induction, expansion, differentiation, and migration. Additionally, signaling pathways that were previously thought to only function in adult stress hematopoiesis, such as inflammatory signaling, have now been revealed to play important roles during HSC formation. Yet, many questions still remain. Recent work in mammalian systems has challenged the hierarchical nature of HSCs claiming that most of steady‐state adult hematopoiesis is maintained by lineage‐restricted and multipotent progenitors once thought to be short‐lived 73, 74. Recently, several groups made the surprising finding that adult tissue‐resident macrophages and microglia are derived from yolk sac cells that arise before definitive HSCs, demonstrating that embryonic cells once thought to be restricted in self‐renewal are rather long‐lived and persist into adulthood 75, 76. Therefore, it is now important to understand the developmental origins and lineal relationships between embryonic HSCs and progenitor populations and these long‐lived adult progenitors. As scientists attempt to make transplantable HSCs in a dish, it is imperative to understand the repertoire and heterogeneity of long‐lived progenitors that an embryo generates. New single‐cell approaches to lineage‐tracing and gene expression profiling in the zebrafish could provide the high resolution required to dissect not only the complexity of hematopoiesis, but also the developing niche that regulates HSC and progenitor cell properties 77 78 79 80.

## Author Contributions

A.D.L.G. and A.S.: conception and design, manuscript writing; T.V.B.: conception and design, financial support, manuscript writing, final approval of manuscript.

## Disclosure of Potential Conflicts of Interest

The authors indicated no potential conflicts of interest.
